# Chronic High Intensity Interval Training (HIIT) exercise in adolescent rats results in cocaine place aversion and ΔFosB induction

**DOI:** 10.1371/journal.pone.0316228

**Published:** 2025-09-17

**Authors:** Nikki Hammond, Nabeel Rahman, Sam Zhan, Mark S. Gold, Kenneth Blum, Teresa Quattrin, Yun Young Yim, Eric J. Nestler, Panayotis K. Thanos

**Affiliations:** 1 Behavioral Neuropharmacology and Neuroimaging Laboratory on Addictions (BNNLA), Research Institute on Addictions, Department of Pharmacology and Toxicology, Jacobs School of Medicine and Biomedical Sciences, University at Buffalo, Buffalo, New York, United States of America; 2 New York College of Podiatric Medicine, New York, New York, United States of America; 3 Department of Psychiatry, Washington University School of Medicine, St. Louis, Missouri, United States of America; 4 Center for Sports, Exercise, and Mental Health, Western University of Health Sciences, Pomona, California, United States of America; 5 Department of Molecular Biology, Adelson School of Medicine, Ariel University, Ariel, Israel; 6 UBMD Pediatrics, JR Oishei Children’s Hospital, University at Buffalo, Buffalo, New York, United States of America; 7 Nash Family Department of Neuroscience and Friedman Brain Institute, Icahn School of Medicine at Mount Sinai, New York, New York, United States of America; 8 Department of Exercise and Nutrition Sciences, University at Buffalo, Buffalo, New York, United States of America; University of Nebraska Medical Center College of Medicine, UNITED STATES OF AMERICA

## Abstract

High-Intensity Interval Training (HIIT) is a form of exercise that has been greatly popularized over the past few years for its many health benefits. Similar to other forms of exercise, HIIT may be beneficial in the prevention of substance use behaviors; however, the extent to which HIIT can impact the reinforcing effects of drugs of abuse during adolescence has not been fully evaluated. Here, we assess the effects of HIIT during adolescence on subsequent cocaine conditioned place preference (CPP) in male Lewis rats. The HIIT exercise exposed rats ran on a treadmill for 30 minutes daily (ten three-minute cycles) for six weeks with progressive speed-increased up to 0.8 mph (21.5m/min), while the sedentary rats remained in their home cage. Following the six weeks of exercise, rats were tested for cocaine (25 mg/kg) CPP. Following completion of the behavior test ∆FosB levels were measured in the brain. Results showed that the HIIT rats showed significantly attenuated place preference (−19%) in their time spent in the cocaine-paired chamber compared to the sedentary environment rats. In addition, HIIT rats had significantly higher (65%) striatum ∆FosB levels compared to the sedentary rats. Our findings show that HIIT exercise during adolescence could be protective against cocaine abuse which may be mediated by an increase in ∆FosB. This finding has important clinical implications with respect to exercise mediated protection against substance misuse and abuse. Future studies will examine this effect in females as well as the potential underlying mechanisms.

## Introduction

In 2019, 23 million individuals ages 12 and up were reported to suffer from a substance misuse disorder [1]. In particular, one of the most frequently abused drugs today is cocaine, ranking second in illegal drug use after cannabis globally [2]. Cocaine primarily increases synaptic dopamine levels by inhibiting dopamine reuptake. Chronic cocaine use results in increased neuronal dendritic branching and spine density in the nucleus accumbens and prefrontal cortex, which is thought to increase the incentive behind drug use [3]. Cocaine abuse also alters the mesolimbic reward pathway in the brain, in part through increased ΔFosB expression in the nucleus accumbens [4].

Previous research has supported the use of exercise for both the prevention and treatment of substance misuse. Exercise has been shown to alter brain metabolism in regions active in the mesolimbic pathway during acute and chronic cocaine use further supporting its indication to aid in substance misuse treatment [5–8]. Physical activity has been shown to decrease cocaine conditioned place preference (CPP), attenuate cocaine cue-induced reinstatement, and inhibit stress-induced reinstatement of cocaine CPP in rodents [9–11], while also reducing cocaine craving and usage in humans [12].

Different exercise regimens display differing levels of efficacy, including varying therapeutic potential regarding neuropsychiatric diseases [13]. High-intensity interval training (HIIT) has been shown to result in greater improvements in VO_2_ max values compared to moderate-intensity continuous exercise (MICT) [14], lowered insulin resistance and decreased fasting blood glucose levels [15], enhanced cognitive performance and working memory capacity [16]. Inactive people are also more likely to continue exercising under a HIIT regimen than MICT [17]. Previous research has shown that MICT during adolescence attenuated future cocaine place preference behavior in females while blocking in males [11], but no such research has been done using HIIT. The present study examined the impact of HIIT treadmill exercise during adolescence on cocaine preference in male rats.

∆FosB, a member of Fos family of proteins, has been found to play a significant role in addictive behaviors that are associated with addiction [18–21]. The ability of ∆FosB to increase sensitivity to drugs of abuse and increase drug seeking behavior has led to it being labeled a sustained molecular switch [21–23]. Areas of the brain in rodents models that have been linked to this drug seeking behavior includes the nucleus accumbens and the dorsal striatum [18,21,23]. Following chronic exposure to cocaine, ∆FosB—but not any other Fos family protein—accumulates and its expression remains stable for weeks [23–26]. This accumulation of ∆FosB is also seen following chronic cocaine self-administration as well as yoked exposure [25,26]. In this paper, we measured the level of ∆FosB expression in the striatum following HIIT and subsequent cocaine place preference with the hypothesis that rats exposed to HIIT exercise would have lower levels compared to sedentary controls.

## Materials and methods

### Animals

Male (n = 32) Lewis rats were obtained at 6 weeks of age (Charles River Laboratories Incorporated). Subjects were housed under standard laboratory conditions (22°C ± 2°C; 12-hour reverse light/dark cycle [lights off: 08:00–20:00]. Food and water were available ad libitum for the duration of the study. Body weights of all subjects were measured daily. This experiment was conducted in accordance with the National Academy of Sciences Guide for the Care and Use of Laboratory Animals (1996) and University at Buffalo Institutional Animal Care and Use Committee (Protocol Number: 202100079).

### Drugs

Cocaine (Sigma-Aldrich, St. Louis, MO, USA) was dissolved in saline at a concentration of 12.5 mg/ml and administered at a dose of 25 mg/kg [11,27].

### Apparatus

#### Treadmill.

**Fig 1:**
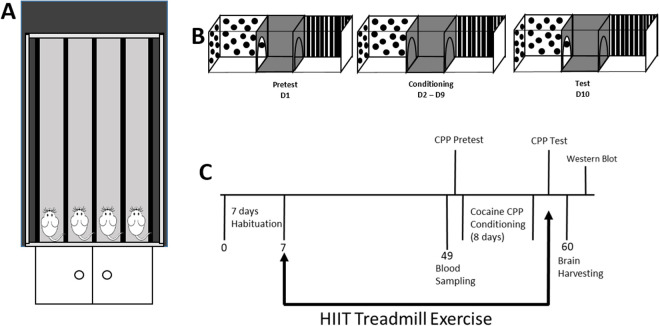
Experimental design A) The treadmill apparatus used for the HIIT daily exercise included four equally spaced Plexiglas lanes. B) Cocaine conditioned place preference timeline. Pretest (Day 1): Free access given to both chambers; drug paired chamber and preferred chamber determined for each subject. Conditioning (Day 2 – Day 9): Subjects confined to saline or cocaine paired chamber on cocaine conditioning days. Test (Day 10): Free access given to both chambers; time spent in cocaine paired chamber measurement. C) The experiment timeline.

A custom-made motorized treadmill was used to conduct forced exercise on the experimental rats (Fig 1A). The treadmill was comprised of four Plexiglas running lanes, each with dimensions of 25 in. x 4.5 in. x 21 in. (L x W x H) [11].

#### Conditioned Place Preference (CPP) boxes.

The CPP apparatus consisted of two compartments connected by a central corridor (Fig 1B). The compartments each possessed dimensions of 12 in. x 7.5 in. x 8 in. (L x W x H). The central corridor possessed dimensions of 4.75 in. x 8.25 in. x 8.25 in. (L x W x H). One compartment consisted of a perforated stainless-steel floor and black and white striped walls. The other compartment consisted of a smooth floor with black and white polka dot walls. The central corridor consisted of a smooth floor with dark gray walls [11].

### Procedure

#### Lab habituation.

After arrival to the laboratory rats underwent one week of habituation during which they had daily handling and body weight measurements (Fig 1C).

#### High Intensity Interval Training (HIIT) exercise regimen.

Subjects were divided into two groups: a sedentary group (n = 16) and a HIIT group (n = 16). Sedentary subjects remained in their home cages for the duration of the study. HIIT subjects were exposed to the chronic exercise regimen (see Fig 1C). Exercise was carried out seven days a week for six weeks. Subjects first underwent five days of habituation to the treadmill at a speed of 0.64 km/h (10 m/min) and for a duration of 10 minutes. Subjects then began the HIIT regimen. Each HIIT session lasted for 30 minutes and consisted of ten three-minute exercise cycles. Each exercise cycle consisted of two minutes of active running followed by one minute of sedentary rest. Running speed began at 0.64 km/h (10 m/min) and was increased by 0.16 km/h (2.68 m/min) every five days until the top speed of 1.29 km/h (21.46 m/min) was reached. Exercise then continued at the top speed for the remainder of the exercise regimen. If needed, an air puff at the end of the treadmill lane was used to maintain running. All exercise sessions were performed during the subjects’ dark cycle (08:00–12:00).

#### Cocaine Conditioned Place Preference (Cocaine CPP).

Two hours after the chronic exercise regimen had been completed, Cocaine CPP was carried out. The CPP procedure consisted of three phases, Pretest, Conditioning, and Test, and spanned for ten days in total. During Pretest (Day 1) subjects were given free access to the entire CPP apparatus for 15 minutes (See Fig 1B). Time spent in each compartment was recorded. The compartment in which more time was spent was defined as the *preferred chamber*, while the compartment in which less time was spent was defined as the *drug-paired chamber*. During Conditioning (Day 2 – Day 9), subjects were given cocaine and saline on an alternating day scheduling, such that cocaine administration was followed by saline administration the following day. Cocaine and saline administration occurred via I.P. injection. Following saline administration, subjects were placed in the *preferred chamber* for 15 minutes. Following cocaine administration, subjects were placed in the *drug-paired chamber* for 15 minutes. Conditioning was carried out for eight days. During Test (Day 10) subjects were once again given free access to the entire CPP apparatus for 15 minutes, as was done during Pretest. Time spent in each compartment was recorded, and Cocaine CPP was determined by comparing time spent in the *drug-paired chamber* on Test Day to time spent in the *drug-paired chamber* during Pretest [11].

#### Stress reactivity testing (Serum Corticosterone ELISA).

Following the end of the HIIT regimen and prior to CPP, subjects’ blood was obtained to assess serum corticosterone levels via an enzyme-linked immunosorbent assay (ELISA). Blood was obtained via tail vein sampling, which was performed as subjects were under light anesthesia (≈ 2.5% isoflurane). Blood was allowed to clot for 30 minutes and was then centrifuged for 15 minutes at 4°C 3000 RPM. Serum was extracted and then stored at ≈ −80°C until being assayed in triplicates for corticosterone using an ELISA (IBL TECAN, Charlotte, North Carolina) according to the manufacturer’s instructions. The CORT ELISA was then ran through a BioTek CYTATION1 Imaging reader. The Imaging reader used the BioTek Gen5 Data Analysis Software to determine the absorbance of each well at 450nm.

#### Tissue preparation (Western blot sampling).

Rats were euthanized 24 hours after CPP test day (Fig 1C). Briefly, rats were euthanized under deep isoflurane anesthesia (~3.0%). Brains were harvested quickly, flash frozen in 2-methlybutane, and stored at −80°C. Bilateral 1 mm tissue punches were taken from the dorsal and ventral striatum in a cryostat at −20°C. Brain punches were weighed and mashed in a 1:40 initial dilution of Pierce^TM^ IP Lysis Buffer (25 mM Tris-HCl pH 7.4, 150 mM NaCl, 1 mM EDTA, 1% NP-40 and 5% glycerol) with Halt^TM^ Protease Inhibitor Cocktail (100X). Homogenates were then spun in a centrifuge for 30 minutes at 4°C 10,000 RPM. Supernatant was then collected and stored in a −80°C freezer.

#### Immunoblot analysis (Western blot).

Aliquots (20 μg of each sample) were ran on an 8–16% polyacrylamide gel for SDS-PAGE and then electrotransferred to nitrocellulose membrane. The blots were blocked twice (30 min per wash) with 0.5% dry milk in TBS-Tween (1X TBS containing 0.1% tween 20) at the room temperature. Then, the blots were washed five times for 5 min each with TBS-Tween (TBST) at room temperature and incubated overnight with FosB anti-rabbit antibody (Cell Signaling, 1:1000) in blocking buffer at 4°C. Next day, the blots were washed five times for 5 min each with TBST and incubated for 1 hr with goat anti-rabbit antibody conjugated to horseradish peroxidase (KPL, 1:10,000) in blocking buffer at room temperature. Then, the blots were washed 3 times for 10 min each with TBST, followed by two additional washes with TBS (15 min per wash). Using Western Lightning™ Chemiluminescence Reagent Plus (Perkin-Elmer) and iBright imager (TheromoFisher), Western blots were imaged. For the β-actin (loading control) detection, the blots were stripped using Restore™ plus Western blot stripping buffer and reblotted as stated above. β-actin anti-mouse antibody (Cell Signaling, 1:2000) and goat anti-mouse antibody conjugated to horseradish peroxidase (KPL, 1:10,000) were used.

### Statistical analysis

To determine the differences between sedentary and HIIT rats, unpaired t-tests were used to assess cocaine CPP, serum corticosterone and ∆FosB levels. Statistical significance for all tests was set to p < 0.05.

## Results

### Cocaine conditioned place preference

**Fig 2:**
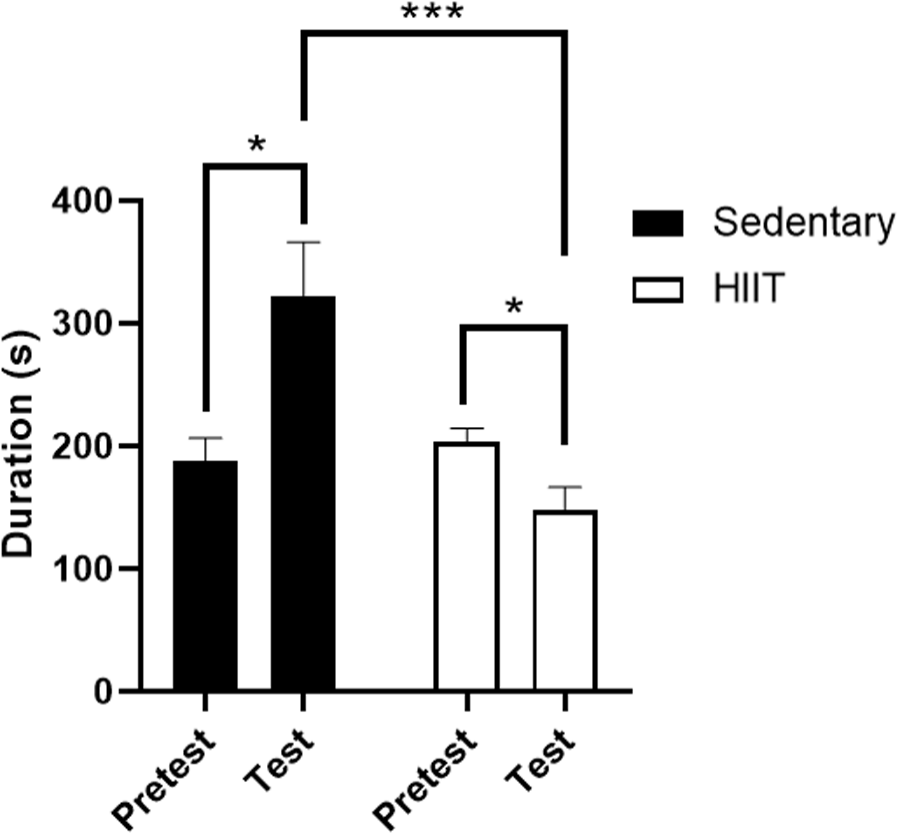
Mean time spent in cocaine chamber (sec + SEM) during Pretest and Test for both Sedentary and HIIT subjects. Sedentary subjects showed a significant preference for cocaine (*p ≤ 0.05). HIIT subjects showed a significant aversion to cocaine (*p ≤ 0.05). Sedentary subjects showed a significantly increased amount of time spent in the cocaine chamber on Test Day compared to that of HIIT subjects on Test Day (*p ≤ 0.001). Time spent in cocaine chamber during Pretest was not statistically significant between Sedentary and HIIT subjects.

All data were analyzed via an unpaired t-test. Sedentary rats exhibited a preference for the cocaine-paired chamber, indicated by a significant increase in time spent in the cocaine chamber from Pretest to Test [t(14)= 2.833, p = 0.0133; Fig 2]. By contrast, the HIIT rats exhibited a place aversion to the cocaine-paired chamber, indicated by a significant decrease in time spent in the cocaine chamber from Pretest to Test [t(28)= 2.576, p = 0.0156; Fig 2]. Sedentary rats thus also showed a significantly increased amount of time spent in the cocaine chamber on Test Day compared to that of the HIIT subjects on Test Day [t(21)= 4.292, p = 0.0003; Fig 2].

### Stress reactivity testing (Serum Corticosterone ELISA)

**Fig 3:**
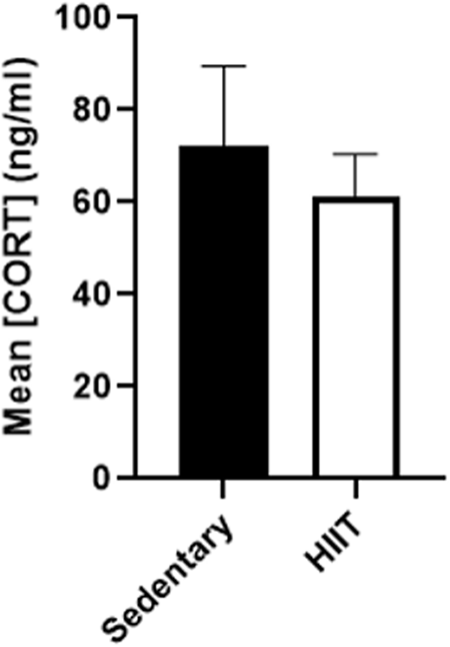
Mean corticosterone (ng/ML + SEM) for both sedentary and HIIT subjects following the chronic exercise regimen and prior to cocaine CPP. Both HIIT and Sedentary subjects showed similar levels of serum corticosterone. A t-test was ran and no significant difference was observed between the two groups (p = 0.6016).

Both the HIIT and the sedentary group of rats were examined for serum corticosterone levels. A T-test revealed [t(10.42)= 0.5384, p = 0.6016] that there was no significant difference between the two groups in terms of serum corticosterone levels (p = ns; Fig 3).

### Immunoblot analysis (∆FosB Western blot)

**Fig 4:**
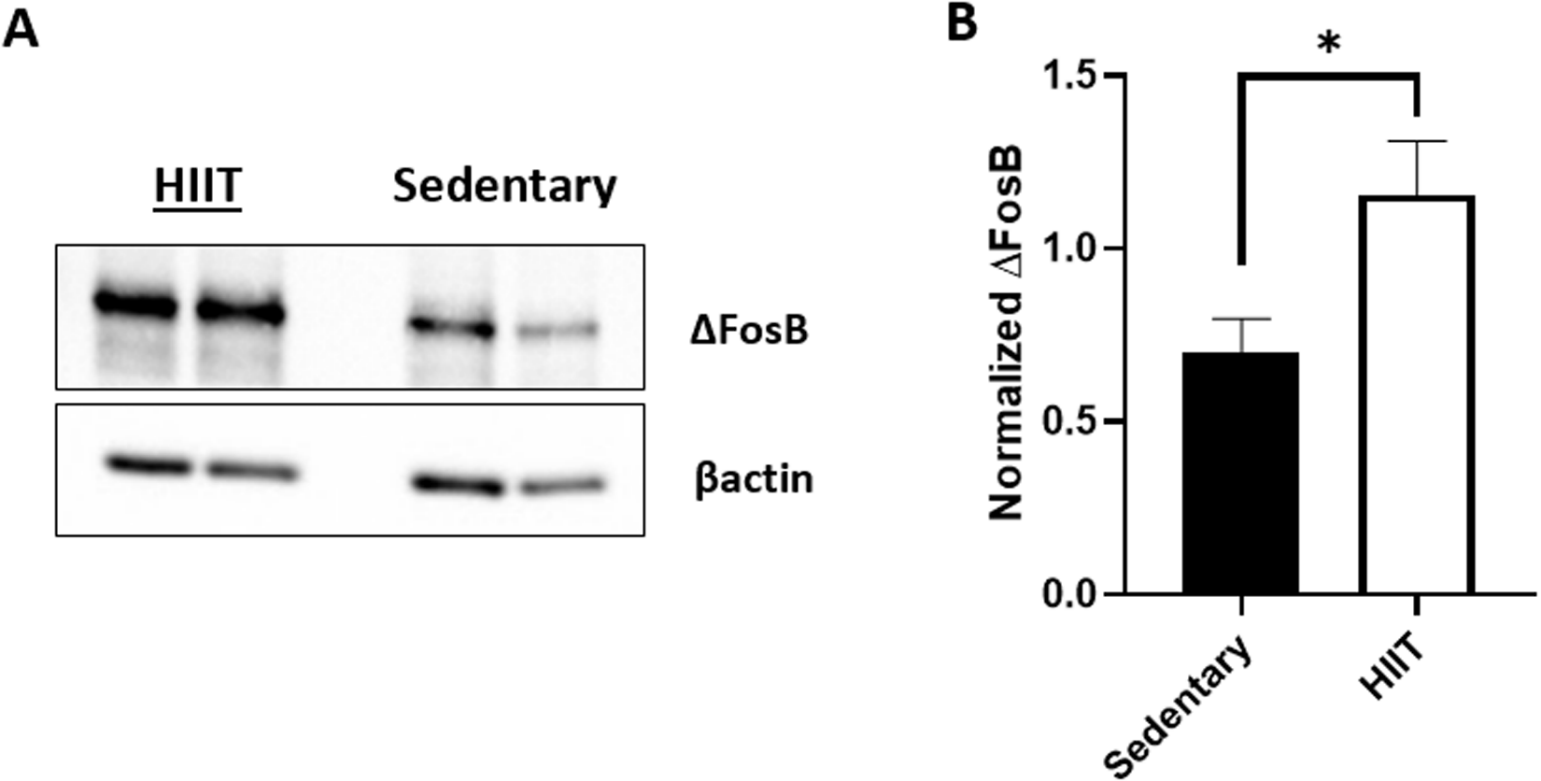
Western blot results of ∆ FosB A) Exemplary western blots of ∆FosB. There are two samples per regimen represented in this figure. FosB anti-rabbit antibody was used to detect ∆FosB in the striatal samples of the sedentary and HIIT animals. B) Normalized ∆FosB (+SEM) for both sedentary (n = 12) and HIIT (n = 18) following the chronic exercise regimen and cocaine CPP. The ∆FosB band was normalized to its corresponding βactin band. The HIIT subjects had significantly higher ∆FosB levels than the sedentary subjects (*p ≤ 0.05).

The dorsal and ventral striatum of the sedentary and HIIT treated rats were analyzed for ∆FosB levels. After running an unpaired t-test it was determined that there was no significant difference in ∆FosB levels between the dorsal and ventral striatum of the sedentary group [t(10)= 1.441, p = 0.1801]. There was also no significant different between the dorsal and ventral striatum of the HIIT group [t(15)= 1.672, p = 0.1153]. Due to this, we combined the dorsal and ventral striatum results and labeled them as striatum. There was a significant increase of ∆FosB in the HIIT compared to the sedentary group [t(28)= 2.184, p = 0.0375; Fig 4].

## Discussion

The present study showed that rats exposed to a chronic HIIT exercise regimen displayed an aversion to cocaine as measured by the cocaine conditioned place preference paradigm. This observed effect of HIIT may be transient due to HIIT exercise being performed during cocaine conditioned place preference. Our lab has previously reported the efficacy of treadmill exercise in decreasing cocaine preference [11]; however, this study exposed rats to chronic moderate-intensity continuous exercise (MICT), commonly regarded as standard aerobic exercise, and showed that exercise-treated rats still displayed a preference for cocaine, yet to a lesser degree [11]. The present study builds on our previous findings by illustrating that HIIT may serve as a more effective intervention in the realm of cocaine abuse than that of MICT by not only decreasing cocaine preference but causing an aversion to cocaine. The same apparatus was used in this study as our previous MICT study. The MICT treadmill running protocol in our previous study consisted of a speed of 10 m/min and a start of 10 min/day [11]. The length of exercise time was increased gradually until 60 min/day of consistent running was achieved [11]. The MICT protocol was ran for a total of 6 weeks which is the same as our current study. The main differences between the two studies exercising protocol is that instead of increasing the amount of time ran we increased the speed to a maximum of 21.46 m/min and the 30 minutes of exercise daily for the animals included breaks. Though this study was not performed to directly compare MICT to HIIT, the question arises as to what physiological and neurological mechanisms underlie the increased efficacy of HIIT compared to MICT relating to cocaine abuse warranting further investigation.

In addressing this question, the first matter to be discussed is the differences between the effects of HIIT and MICT; here, a few general points of difference have been noted. When compared to MICT, HIIT has been shown to result in increased glucose metabolism [11,28], greater reductions in fat mass [11,29], and greater improvements in VO_2_ max [11,14], the lattermost of which is widely used as a measure of cardiorespiratory fitness [11,30]. HIIT has also been shown to possess enhanced efficacy in disease treatment: this phenomenon has been demonstrated with regards to cardiac disease [11,31–33], multiple sclerosis [34], and diabetes [11,35].

The other major matter to be discussed is that of general factors which affect cocaine CPP. It has been shown that the cannabinoid receptor system is implicated in cocaine abuse, with CB1 receptor antagonism and CB2 receptor agonism resulting in reduced cocaine preference [36]. It has previously been shown that MICT does not alter CB1 receptor levels in rats [37]; while HIIT has shown a decrease in CB1 receptor binding in both males and females [38]. It has been shown that the administration of THC increases Fos accumulation in cocaine exposed adolescent rats [39]. As a result, further investigation should be performed to expand upon the endocannabinoid system’s mediation in cocaine preference. Additionally, it has been shown that a neurotensin analog blocks cocaine CPP [40]. Neurotensin is a neuropeptide that has been strongly implicated to act with the dopaminergic system [40]. Previous studies have shown that MICT can increase D1 receptor levels [41] or decreased dopamine D1 receptor levels and increased D2 receptor levels, but this phenomenon has similarly not been explored in HIIT [37,42].

Aside from the aspect of exercise intensity, the role of sex has not been adequately examined with respect to HIIT. Our previous study [11] showed that MICT decreased cocaine preference in females while eliminating cocaine preference in males. In previous studies, 25 mg/kg of cocaine has induced a place preference [11,27]. The present study shows that HIIT not only eliminated cocaine preference in males but caused an aversion to the cocaine-paired environment. As a result, future studies should explore the efficacy of HIIT on cocaine preference in female rats, as our previous findings illustrate sex as a crucial factor. This occurrence is also supported by the literature, as females have been noted to possess higher levels of vulnerability than males during various phases of the addiction process, including acquisition, maintenance, and relapse [43–45]. According to Orihuel *et al.* [39] there were significant sex-dependent interactions between cocaine and adolescent THC exposure in the dorsal hypothalamus, suggesting that cocaine induced a more robust cellular activation in THC exposed females than males. Additionally, it has been shown that when given free access to a running wheel, female rats run significantly more than males [46], thus illustrating a sex difference with regards to exercise. As these factors point to sex differences regarding both exercise and addiction, they highlight the potential value in exploring sex as a factor in the role of exercise on addiction.

HIIT exercise pretreated rats tested for cocaine CPP show higher levels of ∆FosB in their striatum compared to their sedentary animal counterparts. Due to the sub-chronic nature of the cocaine injections, as indicated by the 4 total injections of cocaine provided within a week, this increase in ∆FosB level was caused mostly by the chronic HIIT exercise regimen. Previous studies have shown that when a drug of abuse is administered, there is no increase in ∆FosB level without the functional D1 receptor [47–49]. This indicates that the D1 receptor is needed for ∆FosB expression. HIIT exercise when administered has demonstrated an increase of dopamine type 2-like receptor (D2R) binding in the nucleus accumbens, which has previously been linked to attenuating drug seeking behavior [50]. Males have shown higher levels of D2R binding after HIIT compared to females, making the females more susceptible to addiction [50]. Within the same study there was no significant difference seen in tyrosine hydroxylase or D1 receptor, an explanation for this was possible upper limits to neurotrophic factors due to the healthy nature of the animal subjects [50]. Due to the important role of dopamine signaling in addiction and exercise there is a need for further investigation on how HIIT affects the expression of the D1, D2 and D3 receptors.

As animals exercise, dopamine and ∆FosB levels increase [51,52]. Since ∆FosB has a long half-life [22,53], it is possible that the increase we saw due to HIIT exercise is attenuating or reversing the rewarding dopaminergic effects of cocaine place preference [54,55]. In Zhang *et al.* [56], rats exposed to an enriched environment had higher baseline levels of ∆FosB than their counterparts. After cocaine self-administration, the enriched environment rats did not have a significant increase in ∆FosB. In addition, these rats had reduced cocaine-seeking behavior [56,57]. Since exercise is considered a form of environmental enrichment [58,59], an increase in dopamine from HIIT could be blocking the rewarding sensation of cocaine, thus causing an aversion to the cocaine-paired chamber after undergoing chronic HIIT.

The current study had some limitations. In our analysis of ∆FosB we combined the dorsal and ventral striatum. Though both areas are shown to play a role in substance misuse, there could be differences in expression in both regions making it important for future studies to assess both regions separately when analyzing ∆FosB [21]. All animals used to analyze ∆FosB levels had been exposed to cocaine which in itself has been shown to increase ∆FosB [18,21,23–26]. To make a more direct comparison of the role of HIIT on ∆FosB levels a future study could assess the use of a HIIT control group which do not undergo cocaine testing. This study only used male subjects to study the use of HIIT to attenuate cocaine CPP. Future studies need to also test this in females [11].

## Conclusion

The present study showed that HIIT exercise during adolescence effectively prevented cocaine preference compared to control rats in adulthood. Specifically, HIIT exercise in adolescent rats produced a significant aversion to the cocaine-paired environment. These novel results encourage and further support the benefit of HIIT in reducing the risk of substance-related behaviors, such as cocaine preference. These result may represent the transient effect of HIIT with further investigation required to investigate the long term implications of performing HIIT. Future research will further explore the underlying mechanisms behind this phenomenon of HIIT exercise on ∆FosB and substance misuse. Finally, these findings support the concept of specific exercise dosing regimens like HIIT having distinct effects on drug abuse behavior mediated by ∆FosB and could have important future implications for a personalized medicine approach to drug abuse intervention.

## Supporting information

S1 Fig Original unedited western blot gels. Original western blots were taken in 3 batches with individual samples blocked twice on the same gel. Average values for ∆FosB (37 kDa) were calculated per experimental group (refer to figure 4 in main text). β-actin (kDa) was used as the load control for all blots.
(PDF)

S2 Dataset A. Dataset – Cocaine Conditioned Place Preference (CPP). Dataset of the time spent in the cocaine chamber in the pretest and test run of cocaine CPP with the outliers removed. This data was used in our statistical analysis and subsequently used in our graph. **B. Dataset** – **Serum Corticosterone ELISA.** Dataset of the serum corticosterone levels prior to cocaine CPP and after treadmill running with the outliers removed. This data was used in our statistical analysis and subsequently used in our graph. **C. Dataset** – **Normalized ∆FosB.** Dataset of normalized ∆FosB with the outliers removed. This data was used in our statistical analysis and subsequently used in our graph.
(PDF)

## References

[1] Nikolovska H. 42 Addiction Statistics and Facts to Know in 2019. [online]. DisturbMeNot: 2019 [Acessed date 10 September 2019]. Available from: https://disturbmenot.co/addiction-statistics/

[2] Karila L, Zarmdini R, Petit A, Lafaye G, Lowenstein W, Reynaud M. Addiction à la cocaïne: données actuelles pour le clinicien. La Presse Médicale. 2014;43(1):9–17. 23727012 10.1016/j.lpm.2013.01.069

[3] Volkow ND, Li T-K. Drug addiction: the neurobiology of behaviour gone awry. Nat Rev Neurosci. 2004;5(12):963–70. doi: 10.1038/nrn1539 15550951

[4] Kelz MB, Chen J, Carlezon WA Jr, Whisler K, Gilden L, Beckmann AM, et al. Expression of the transcription factor deltaFosB in the brain controls sensitivity to cocaine. Nature. 1999;401(6750):272–6. doi: 10.1038/45790 10499584

[5] Hanna C, Hamilton J, Arnavut E, Blum K, Thanos PK. Brain mapping the effects of chronic aerobic exercise in the rat brain using FDG PET. J Pers Med. 2022;12(6):860. doi: 10.3390/jpm12060860 35743644 PMC9224807

[6] Hanna C, Hamilton J, Blum K, Badgaiyan RD, Thanos PK. Exercise modulates brain glucose utilization response to acute cocaine. J Pers Med. 2022;12(12):1976. doi: 10.3390/jpm12121976 36556197 PMC9788493

[7] Hanna C, Yao R, Sajjad M, Gold M, Blum K, Thanos PK. Exercise modifies the brain metabolic response to chronic cocaine exposure inhibiting the stria terminalis. Brain Sci. 2023;13(12):1705. doi: 10.3390/brainsci13121705 38137153 PMC10742065

[8] Powell A, Hanna C, Sajjad M, Yao R, Blum K, Gold MS, et al. Exercise influences the brain’s metabolic response to chronic cocaine exposure in male rats. J Pers Med. 2024;14(5):500. doi: 10.3390/jpm14050500 38793082 PMC11122626

[9] Robison LS, Alessi L, Thanos PK. Chronic forced exercise inhibits stress-induced reinstatement of cocaine conditioned place preference. Behav Brain Res. 2018;353:176–84. 30036547 10.1016/j.bbr.2018.07.009 PMC6319952

[10] Thanos PK, Stamos J, Robison LS, Heyman G, Tucci A, Wang G-J, et al. Daily treadmill exercise attenuates cocaine cue-induced reinstatement and cocaine induced locomotor response but increases cocaine-primed reinstatement. Behav Brain Res. 2013;239:8–14. doi: 10.1016/j.bbr.2012.10.035 23103403 PMC3596018

[11] Thanos PK, Tucci A, Stamos J, Robison LS, Wang GJ, Anderson BJ. Chronic forced exercise during adolescence decreases cocaine conditioned place preference in Lewis rats. Behav Brain Res. 2010;215(1):77–82. 20615434 10.1016/j.bbr.2010.06.033 PMC3188407

[12] De La Garza R, Yoon JH, Thompson-Lake DGY, Haile CN, Eisenhofer JD, Newton TF, et al. Treadmill exercise improves fitness and reduces craving and use of cocaine in individuals with concurrent cocaine and tobacco-use disorder. Psychiatry Res. 2016;245:133–40. 27541349 10.1016/j.psychres.2016.08.003 PMC5067203

[13] Swenson S, Blum K, McLaughlin T, Gold MS, Thanos PK. The therapeutic potential of exercise for neuropsychiatric diseases: a review. J Neurol Sci. 2020;412:116763. doi: 10.1016/j.jns.2020.116763 32305746

[14] Milanović Z, Sporiš G, Weston M. Effectiveness of High-Intensity Interval Training (HIT) and continuous endurance training for VO2max improvements: a systematic review and meta-analysis of controlled trials. Sports Med. 2015;45(10):1469–81. doi: 10.1007/s40279-015-0365-0 26243014

[15] Jelleyman C, Yates T, O’Donovan G, Gray LJ, King JA, Khunti K, et al. The effects of high-intensity interval training on glucose regulation and insulin resistance: a meta-analysis. Obes Rev. 2015;16(11):942–61. doi: 10.1111/obr.12317 26481101

[16] Moreau D, Kirk IJ, Waldie KE. High-intensity training enhances executive function in children in a randomized, placebo-controlled trial. eLife. 2017;6. 10.7554/eLife.25062 PMC5566451 28825973

[17] Epstein LH, O’Donnell S, Biondolillo MJ, Hostler D, Roemmich JN. Comparing the reinforcing value of High Intensity Interval Training versus moderate intensity aerobic exercise in sedentary adults. Physiol Behav. 2021;238:113468. doi: 10.1016/j.physbeh.2021.113468 34033846 PMC9667389

[18] Harris GC, Hummel M, Wimmer M, Mague SD, Aston-Jones G. Elevations of FosB in the nucleus accumbens during forced cocaine abstinence correlate with divergent changes in reward function. Neuroscience. 2007;147(3):583–91. doi: 10.1016/j.neuroscience.2007.04.050 17560044 PMC1986745

[19] Hope B, Kosofsky B, Hyman SE, Nestler EJ. Regulation of immediate early gene expression and AP-1 binding in the rat nucleus accumbens by chronic cocaine. Proc Natl Acad Sci U S A. 1992;89(13):5764–8. doi: 10.1073/pnas.89.13.5764 1631058 PMC402098

[20] Hope BT, Nye HE, Kelz MB, Self DW, Iadarola MJ, Nakabeppu Y, et al. Induction of a long-lasting AP-1 complex composed of altered Fos-like proteins in brain by chronic cocaine and other chronic treatments. Neuron. 1994;13(5):1235–44. doi: 10.1016/0896-6273(94)90061-2 7946359

[21] Nestler EJ. Transcriptional mechanisms of addiction: role of ΔFosB. Philos Trans R Soc B Biol Sci. 2008;363(1507):3245–55. 10.1098/rstb.2008.0067 PMC2607320 18640924

[22] Nestler E. The neurobiology of cocaine addiction. Sci Prac Pers. 2005;3(1):4–10. 10.1151/spp05314 PMC2851032 18552739

[23] Nestler EJ, Barrot M, Self DW. DeltaFosB: a sustained molecular switch for addiction. Proc Natl Acad Sci U S A. 2001;98(20):11042–6. doi: 10.1073/pnas.191352698 11572966 PMC58680

[24] Chocyk A, Czyrak A, Wedzony K. Acute and repeated cocaine induces alterations in FosB/DeltaFosB expression in the paraventricular nucleus of the hypothalamus. Brain Res. 2006;1090(1):58–68. doi: 10.1016/j.brainres.2006.03.045 16674926

[25] Larson EB, Akkentli F, Edwards S, Graham DL, Simmons DL, Alibhai IN, et al. Striatal regulation of ΔFosB, FosB, and cFos during cocaine self-administration and withdrawal. J Neurochem. 2010;115(1):112–22. doi: 10.1111/j.1471-4159.2010.06907.x 20633205 PMC2939959

[26] Perrotti LI, Weaver RR, Robison B, Renthal W, Maze I, Yazdani S, et al. Distinct patterns of DeltaFosB induction in brain by drugs of abuse. Synapse. 2008;62(5):358–69. doi: 10.1002/syn.20500 18293355 PMC2667282

[27] Bobzean SAM, Dennis TS, Addison BD, Perrotti LI. Influence of sex on reinstatement of cocaine-conditioned place preference. Brain Res Bull. 2010;83(6):331–6. doi: 10.1016/j.brainresbull.2010.09.003 20851744

[28] Laursen PB, Jenkins DG. The scientific basis for high-intensity interval training. Sports Med. 2012;32(1):53–73. 10.2165/00007256-200232010-00003 11772161

[29] Zhang H, Tong TK, Qiu W, Zhang X, Zhou S, Liu Y, et al. Comparable effects of high-intensity interval training and prolonged continuous exercise training on abdominal visceral fat reduction in obese young women. J Diabetes Res. 2017;2017:5071740. doi: 10.1155/2017/5071740 28116314 PMC5237463

[30] Ross R, Blair SN, Arena R, Church TS, Després JP, Franklin BA, et al. Importance of assessing cardiorespiratory fitness in clinical practice: a case for fitness as a clinical vital sign: a scientific statement from the American Heart Association. Circulation. 2016;134(24). 10.1161/CIR.0000000000000461 27881567

[31] Hussain SR, Macaluso A, Pearson SJ. High-Intensity interval training versus moderate-intensity continuous training in the prevention/management of cardiovascular disease. Cardiol Rev. 2016;24(6):273–81. doi: 10.1097/CRD.0000000000000124 27548688

[32] Ito S, Mizoguchi T, Saeki T. Review of high-intensity interval training in cardiac rehabilitation. Internal Med. 2016;55(17):2329–36. 27580530 10.2169/internalmedicine.55.6068

[33] Ribeiro PAB, Boidin M, Juneau M, Nigam A, Gayda M. High-intensity interval training in patients with coronary heart disease: prescription models and perspectives. Ann Phys Rehabil Med. 2017;60(1):50–7. 27346629 10.1016/j.rehab.2016.04.004

[34] Campbell E, Coulter EH, Paul L. High Intensity Interval Training for people with multiple sclerosis: a systematic review. Mult Scler Relat Disord. 2018;24:55–63. doi: 10.1016/j.msard.2018.06.005 29936326

[35] Wormgoor SG, Dalleck LC, Zinn C, Harris NK. Effects of high-intensity interval training on people living with type 2 diabetes: a narrative review. Can J Diabetes. 2017;41(5):536–47. doi: 10.1016/j.jcjd.2016.12.004 28366674

[36] Delis F, Polissidis A, Poulia N, Justinova Z, Nomikos GG, Goldberg SR, et al. Attenuation of cocaine-induced conditioned place preference and motor activity via cannabinoid CB2 receptor agonism and CB1 receptor antagonism in rats. Int J Neuropsychopharmacol. 2017;20(3):269–78. doi: 10.1093/ijnp/pyw102 27994006 PMC5408977

[37] Swenson S, Hamilton J, Robison L, Thanos PK. Chronic aerobic exercise: lack of effect on brain CB1 receptor levels in adult rats. Life Sci. 2019;230:84–8. doi: 10.1016/j.lfs.2019.05.058 31128137

[38] Tyler J, Park Y, Lu H, Roeder N, Richardson B, Gold MS, et al. High-intensity interval training exercise decreases brain CB1 receptor levels in male and female adult rats. Neuroscience. 2025;573:254–63. 40122443 10.1016/j.neuroscience.2025.03.038

[39] Orihuel J, Gómez-Rubio L, Valverde C, Capellán R, Roura-Martínez D, Ucha M, et al. Cocaine-induced Fos expression in the rat brain: Modulation by prior Δ9-tetrahydrocannabinol exposure during adolescence and sex-specific effects. Brain Res. 2021;1764:147480. doi: 10.1016/j.brainres.2021.147480 33861997

[40] Boules M, Netz R, Fredrickson PA, Richelson E. A neurotensin analog blocks cocaine-conditioned place preference and reinstatement. Behav Pharmacol. 2016;27(2-3 Spec Issue):236–9. doi: 10.1097/FBP.0000000000000227 26901162

[41] Bobick M, Hanna C, Tyler J, Mohr P, Lu H, Powell A, et al. Exercise leads to brain glucose metabolism activation, increased dopamine D1 receptor levels and is negatively correlated with social behavior. J Integr Neurosci. 2025;24(6):36646. doi: 10.31083/JIN36646 40613367

[42] Robison LS, Swenson S, Hamilton J, Thanos PK. Exercise reduces dopamine D1R and increases D2R in rats: implications for addiction. Med Sci Sports Exerc. 2018;50(8):1596–602. doi: 10.1249/MSS.0000000000001627 29613999

[43] Lynch W, Roth M, Carroll M. Biological basis of sex differences in drug abuse: preclinical and clinical studies. Psychopharmacology. 2002;164(2):121–37. 12404074 10.1007/s00213-002-1183-2

[44] Ahmed R, Zyla S, Hammond N, Blum K, Thanos PK. The role of estrogen signaling and exercise in drug abuse: a review. Clin Pract. 2024;14(1):148–63. 38248436 10.3390/clinpract14010012 PMC10801537

[45] Perreault B, Hammond N, Thanos PK. Effects of exercise on testosterone and implications of drug abuse: a review. Clin Neuropharmacol. 2023;46(3):112–22. doi: 10.1097/WNF.0000000000000546 37191565

[46] Eikelboom R, Mills R. A microanalysis of wheel running in male and female rats. Physiol Behav. 1988;43(5):625–30. doi: 10.1016/0031-9384(88)90217-x 3200918

[47] Lazenka MF, Tomarchio AJ, Lichtman AH, Greengard P, Flajolet M, Selley DE, et al. Role of dopamine type 1 receptors and dopamine- and cAMP-regulated phosphoprotein Mr 32 kDa in Δ9-Tetrahydrocannabinol–mediated induction of ΔFosB in the mouse forebrain. J Pharmacol Exp Ther. 2015;354(3):316–27. 26099530 10.1124/jpet.115.224428 PMC4538878

[48] Muller DL, Unterwald EM. D1 dopamine receptors modulate deltaFosB induction in rat striatum after intermittent morphine administration. J Pharmacol Exp Ther. 2005;314(1):148–54. doi: 10.1124/jpet.105.083410 15772255

[49] Zhang D, Zhang L, Lou DW, Nakabeppu Y, Zhang J, Xu M. The dopamine D1 receptor is a critical mediator for cocaine-induced gene expression. J Neurochem. 2002;82(6):1453–64. doi: 10.1046/j.1471-4159.2002.01089.x 12354293

[50] Tyler J, Podaras M, Richardson B, Roeder N, Hammond N, Hamilton J, et al. High Intensity Interval Training exercise increases dopamine D2 levels and modulates brain dopamine signaling. Front Public Health. 2023. 10.3389/fpubh.2023.1257629 PMC10773799 38192549

[51] Arnold MR, Greenwood BN, McArthur JA, Clark PJ, Fleshner M, Lowry CA. Effects of repeated voluntary or forced exercise on brainstem serotonergic systems in rats. Behav Brain Res. 2020;378:112237. doi: 10.1016/j.bbr.2019.112237 31525404 PMC6936333

[52] Meeusen R, Piacentini MF, De Meirleir K. Brain microdialysis in exercise research. Sports Med. 2001;31(14):965–83. 11735681 10.2165/00007256-200131140-00002

[53] Lobo MK, Nestler EJ. The striatal balancing act in drug addiction: distinct roles of direct and indirect pathway medium spiny neurons. Front Neuroanatomy. 2011;5:41. 10.3389/fnana.2011.00041 PMC3140647 21811439

[54] Bilsky E, Montegut M, Nicholas M, Reid L. CGS 10746B, a novel dopamine release inhibitor, blocks the establishment of cocaine and MDMA conditioned place preferences. Pharmacol Biochem Behav. 1998;59(1):215–20. 9443558 10.1016/s0091-3057(97)00424-3

[55] Galaj E, Manuszak M, Arastehmanesh D, Ranaldi R. Microinjections of a dopamine D1 receptor antagonist into the ventral tegmental area block the expression of cocaine conditioned place preference in rats. Behav Brain Res. 2014;272:279–85. doi: 10.1016/j.bbr.2014.07.008 25017572

[56] Zhang Y, Crofton EJ, Li D, Lobo MK, Fan X, Nestler EJ, et al. Overexpression of DeltaFosB in nucleus accumbens mimics the protective addiction phenotype, but not the protective depression phenotype of environmental enrichment. Front Behav Neurosci. 2014;8:297. doi: 10.3389/fnbeh.2014.00297 25221490 PMC4148937

[57] Green TA, Alibhai IN, Roybal CN, Winstanley CA, Theobald DEH, Birnbaum SG, et al. Environmental enrichment produces a behavioral phenotype mediated by low cyclic adenosine monophosphate response element binding (CREB) activity in the nucleus accumbens. Biol Psychiatry. 2010;67(1):28–35. doi: 10.1016/j.biopsych.2009.06.022 19709647 PMC2860655

[58] Kempermann G. Environmental enrichment, new neurons and the neurobiology of individuality. Nat Rev Neurosci. 2019;20(4):235–45. doi: 10.1038/s41583-019-0120-x 30723309

[59] Xie H, Wu Y, Jia J, Liu G, Zhang Q, Yu K. Enrichment-induced exercise to quantify the effect of different housing conditions: A tool to standardize enriched environment protocols. Behav Brain Res. 2013;249:81–9. 23624191 10.1016/j.bbr.2013.04.032

