# Supplementary material for: Chronic High Intensity Interval Training (HIIT) exercise in adolescent rats results in cocaine place aversion and ΔFosB induction
Source: PLoS One. 2025 Sep 17;20(9):e0316228. doi: 10.1371/journal.pone.0316228 (PMC12443240; doi:10.1371/journal.pone.0316228)
Supplement: S1 Fig — Original western blots were taken in 3 batches with individual samples blocked twice on the same gel. Average values for ∆FosB (37 kDa) were calculated per experimental group (refer to figure 4 in main text). β-actin (kDa) was used as the load control for all blots. (PDF) [file pone.0316228.s001.pdf]

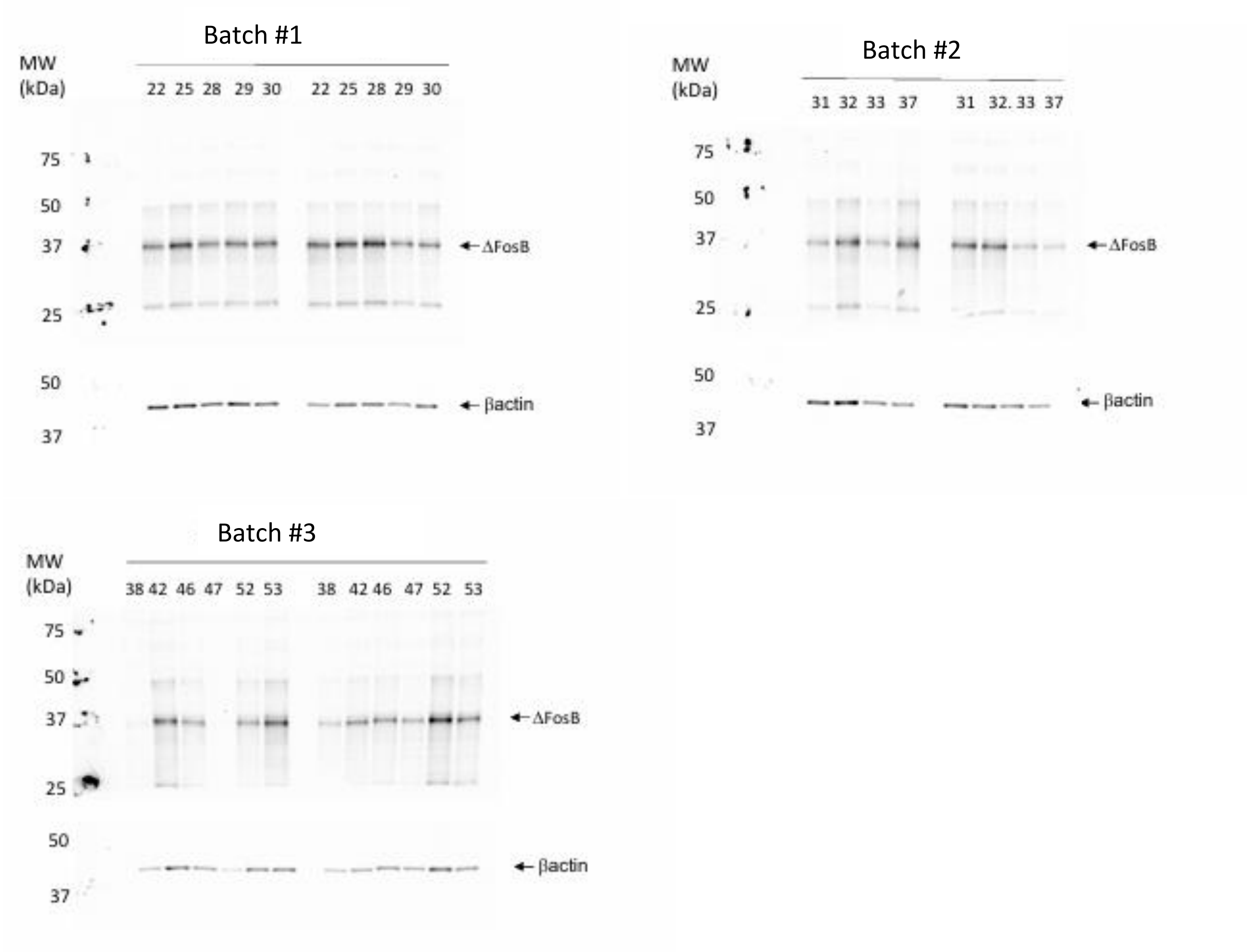

S1 Fig. Original unedited western blot gels. Original western blots were taken in 3 batches with individual samples blocked twice on the same gel. Average values for ΔFosB (37 kDa) were calculated per experimental group (refer to figure 4 in main text). β-actin (kDa) was used as the load control for all blots.
