# Supplementary material for: Chronic High Intensity Interval Training (HIIT) exercise in adolescent rats results in cocaine place aversion and ΔFosB induction
Source: PLoS One. 2025 Sep 17;20(9):e0316228. doi: 10.1371/journal.pone.0316228 (PMC12443240; doi:10.1371/journal.pone.0316228)
Supplement: S2 Dataset — Dataset of the time spent in the cocaine chamber in the pretest and test run of cocaine CPP with the outliers removed. This data was used in our statistical analysis and subsequently used in our graph. B. Dataset – Serum Corticosterone ELISA. Dataset of the serum corticosterone levels prior to cocaine CPP and after treadmill running with the outliers removed. This data was used in our statistical analysis and subsequently used in our graph. C. Dataset – Normalized ∆FosB. Dataset of normalized ∆FosB with the outliers removed. This data was used in our statistical analysis and subsequently used in our graph. (PDF) [file pone.0316228.s002.pdf]

	Sedentary pretest	Sedentary test	HIIT Pretest	HIIT Test
Duration (s)	120.90	552.25	249.70	98.50
	191.15	226.74	250.63	76.49
	181.03	230.84	279.13	242.50
	157.25	228.62	179.26	117.82
	211.46	259.93	196.54	150.57
	268.00	341.76	203.59	82.24
	250.17	272.58	171.20	154.26
	116.74	466.95	231.92	145.81
			146.67	103.89
			221.20	52.36
			182.19	121.06
			120.27	170.38
			214.05	282.96
			217.11	295.57
			191.58	120.37

S2A Dataset. Cocaine Conditioned Place Preference (CPP). Dataset of the time spent in the cocaine chamber in the pretest and test run of cocaine CPP with the outliers removed. This data was used in our statistical analysis and subsequently used in our graph.

	HIIT	Sedentary
CORT (ng/ml)	62.21	111.10
	90.09	160.70
	83.64	100.00
	27.03	12.00
	68.62	22.90
	87.30	42.00
	37.82	58.20
	34.40	69.20

S2B Dataset. Serum Corticosterone ELISA. Dataset of the serum corticosterone levels prior to cocaine CPP and after treadmill running with the outliers removed. This data was used in our statistical analysis and subsequently used in our graph.

Normalized ΔFosB	Sedentary	HIIT
	0.132	0.731
	0.827	1.106
	0.789	0.908
	0.024	0.839
	0.545	1.350
	1.064	0.388
	0.545	0.747
	0.986	2.425
	0.811	1.521
	0.631	2.254
	1.000	1.500
	1.036	0.864
		0.816
		1.226
		0.504
		0.469

S2C Dataset. Normalized ΔFosB. Dataset of normalized ΔFosB with the outliers removed. This data was used in our statistical analysis and subsequently used in our graph.
